# Peri-implant soft tissue health: Single-piece implants versus conventional bridges

**DOI:** 10.6026/973206300221546

**Published:** 2026-03-31

**Authors:** Pritha Negi, Ravindra Babu Lakkaraju, Sukumar Ragutla, Parekh Parth Tushar, Harikrishnan Balachandran Pillai, Lahari Priya Singareddy

**Affiliations:** 1 Department of Prosthodontics and Crown & Bridge, K. M. Shah Dental College and Hospital, Sumandeep Vidyapeeth, Vadodara, Gujarat, India; 2 Department of Dental Surgery, Government Medical College, Quthbullapur, Medchal Malkajgiri, Telangana, India; 3 Department of Prosthodontics and Implantology, Tirumala Institute of Dental Sciences and Research Centre, Nizamabad, Telangana, India; 4 Department of Oral and Maxillofacial Surgery, Karnavati School of Dentistry, Karnavati University, Gandhinagar, Gujarat, India; 5 Department of Periodontology, Azeezia College of Dental Sciences and Research, Kollam, Kerala, India; 6 Department of Public Health, St. Francis College, New York City, New York, USA

**Keywords:** Dental implants, peri-implantitis, soft tissue health, bone remodeling, prosthodontics

## Abstract

Evidence directly comparing peri-implant soft tissue health around single-piece implants and natural abutment teeth in conventional fixed bridges remains limited. Therefore, it is of interest to evaluate peri-implant soft tissue parameters around single-piece implants versus abutment teeth supporting fixed partial dentures. Standardized clinical and radiographic protocols were used to measure gingival index, probing depth, bleeding on probing and marginal bone levels over 12 months. Single-piece implants demonstrated lower probing depths, reduced bleeding scores and less marginal bone loss compared with abutment teeth. Both groups maintained clinically acceptable tissue stability throughout follow-up. Thus, we show that single-piece implants achieve favourable peri-implant soft tissue health comparable or superior to conventional bridge abutments.

## Background:

Dental implants provide a reliable method for replacing missing teeth. Long-term success depends on stable peri-implant soft tissue and marginal bone. Inflammatory changes in peri-implant mucosa can progress to peri-implantitis and compromise implant survival [1]. Single-piece implants integrate the implant and abutment into one unit. This configuration eliminates the implant-abutment microgap. The microgap is a recognized niche for bacterial colonization and inflammatory infiltration [2]. Its elimination may enhance mucosal sealing and tissue stability. Conventional fixed partial dentures depend on prepared natural abutment teeth. Sub gingival finish lines and cement remnants can encourage plaque retention and gingival irritation [3]. These factors may lead to periodontal inflammation and attachment loss. Peri-implant and periodontal soft tissue health is evaluated using gingival index, probing depth, bleeding on probing and marginal bone level. Stable values indicate an intact biological barrier, while deviations suggest early inflammatory changes [4]. Previous comparisons between one-piece and two- piece implants report minimal long-term differences in bone behavior [5]. However, direct clinical evidence comparing single-piece implants with natural abutment teeth supporting bridges remains limited. Such data are required for evidence-based selection of tooth replacement strategies. Therefore, it is of interest to evaluate peri-implant soft tissue health around single-piece implants and periodontal tissue around bridge abutments under standardized clinical conditions.

## Materials and Methods:

## Study design and population:

This prospective comparative clinical study was conducted on 40 systemically healthy patients (22 males, 18 females; mean age 45 ± 6 years) who required single missing tooth replacement in posterior mandibular or maxillary regions. Ethical approval was obtained prior to study initiation.

## Participants were divided into two groups:

[1] Group I: 40 single-piece implants (Adin™ system) placed in healed ridges.

[2] Group II: 40 abutment teeth supporting conventional fixed partial dentures (fpds).

## Inclusion and exclusion criteria:

Inclusion criteria were good oral hygiene (OHI-S ≤ 1.5), sufficient bone volume (≥6 mm width) and keratinized gingiva ≥2 mm. Exclusion criteria included uncontrolled diabetes, smoking, bruxism and untreated periodontitis.

## Clinical and radiographic evaluation:

The following parameters were assessed at baseline, 3, 6 and 12 months post-loading:

[1] Modified Gingival Index (mgi) - Löe and Silness criteria

[2] Probing Pocket Depth (PPD) - measured at 4 sites using a UNC-15 probe

[3] Bleeding on Probing (BOP) - dichotomous score

[4] Marginal Bone Level (MBL) - measured radiographically from implant shoulder or CEJ to alveolar crest

## Statistical analysis:

Data were analyzed using SPSS v26. Intergroup comparisons were performed with the independent t-test and intragroup changes over time were assessed by repeated measures ANOVA. A P < 0.05 was considered statistically significant.

## Results:

At 12 months, analysis of peri-implant and abutment sites Table 1 (see PDF) revealed that single-piece implants demonstrated better soft tissue health compared with abutment teeth in conventional bridges. The mean gingival index was slightly lower in implants (0.72 ± 0.31) than abutment teeth (0.86 ± 0.36), though the difference was not statistically significant (P = 0.12). However, probing pocket depth, bleeding on probing and marginal bone loss were significantly lower around implants (2.43 ± 0.54 mm, 12.5%, 0.82 ± 0.27 mm) compared to abutment teeth (2.89 ± 0.67 mm, 16.8%, 1.12 ± 0.39 mm), indicating superior peri-implant tissue stability (P < 0.05) Table 1 (see PDF). Table 2 (see PDF) shows temporal changes in peri-implant parameters within the implant group. A gradual increase in gingival index and probing depth was observed from baseline to 12 months, signifying normal tissue maturation without inflammatory breakdown. Marginal bone loss increased slightly from baseline (0.00 mm) to 12 months (0.82 ± 0.27 mm), representing expected early remodeling rather than pathological loss (P < 0.05). These findings confirm that single-piece implants maintain healthy peri-implant tissues with minimal crestal bone alterations during the first year of function Table 2 (see PDF).

## Discussion:

The present study compared peri-implant soft tissue conditions around single-piece dental implants with periodontal tissue status around natural abutment teeth supporting conventional fixed partial dentures. The findings demonstrated that implants exhibited lower probing pocket depths, reduced bleeding on probing, and less marginal bone loss compared with abutment teeth after 12 months of functional loading. These observations suggest that single-piece implant systems may provide favourable biological conditions for maintaining peri- implant tissue stability [6, 7]. Marginal bone preservation is widely considered one of the primary indicators of long-term implant success. In the present study, mean marginal bone loss around implants at 12 months was 0.82 ± 0.27 mm, which falls within the limits of physiologic remodeling reported in clinical studies [8, 9]. Rathe *et al.* investigated the influence of abutment emergence angle and restorative design on peri-implant bone stability and reported that bone levels remained largely stable in implants with favourable prosthetic parameters, indicating that implant geometry and prosthetic emergence profile can significantly influence crestal bone maintenance [10]. Similarly, Betha *et al.* observed that appropriate implant placement and prosthetic loading protocols contribute to stable peri-implant bone levels during early follow-up periods [11]. The bone changes observed in the present study therefore appear consistent with expected biological remodeling rather than pathological bone loss. The structural design of single-piece implants may also explain the favourable peri-implant tissue responses observed in this investigation. Unlike conventional two-piece systems, single-piece implants eliminate the implant-abutment interface, thereby removing the microgap that may act as a reservoir for bacterial colonization. Sala *et al.* reported that prosthetic connection characteristics can influence microbial accumulation and the inflammatory status of peri-implant tissues [12]. Likewise, Walter *et al.* demonstrated that implant-abutment interfaces may contribute to bacterial penetration and peri-implant mucosal inflammation when microgaps are present [13]. The absence of this interface in single-piece implants may therefore enhance mucosal sealing and contribute to improved peri-implant soft tissue stability. Probing pocket depth is another essential clinical parameter used to evaluate peri-implant tissue health. In the present study, mean probing depths around implants remained within physiologic limits and were significantly lower than those recorded around bridge abutment teeth.

Borgonovo *et al.* reported that probing depths of approximately 2-3 mm around implants are commonly associated with healthy peri-implant mucosa and stable connective tissue attachment [14]. Similarly, Coli and Jemt found that successful implant restorations generally demonstrate stable peri-implant probing depths during early functional loading, reflecting favourable osseointegration and soft tissue adaptation [15]. The probing depth values recorded in the present study therefore indicate healthy peri-implant mucosal conditions during the one-year observation period. Bleeding on probing is widely regarded as a sensitive indicator of peri-implant mucosal inflammation. In the current study, implants showed significantly lower bleeding scores compared with abutment teeth supporting bridges. Fernandes *et al.* described peri- implant diseases as inflammatory conditions triggered primarily by local microbial biofilm accumulation around implant surfaces and prosthetic components [16]. In addition, Steyer *et al.* reported that plaque accumulation and inadequate oral hygiene are major contributors to peri-implant mucositis and bleeding on probing [17]. Therefore, the lower bleeding scores observed around implants in this study may reflect favourable prosthetic contours and improved accessibility for plaque control compared with conventional bridge restorations. In contrast, abutment teeth supporting fixed partial dentures demonstrated slightly higher gingival inflammation parameters. This may be related to plaque accumulation at crown margins or the presence of residual luting cement, both of which have been identified as potential risk factors for periodontal inflammation around prosthetic restorations. Bonfanti-Gris *et al.* emphasized that prosthetic design characteristics, including restoration margins and cement interfaces, may influence peri-implant and periodontal tissue responses [18]. Similarly, Wimmer *et al.* reported that prosthetic factors and plaque accumulation play an important role in the development of inflammatory changes in peri-implant tissues and surrounding periodontal structures [19]. The biological importance of maintaining healthy peri-implant mucosa has been emphasized in several studies. Healthy peri-implant mucosa forms a protective barrier that limits microbial infiltration and helps maintain implant stability. Pereira *et al.* highlighted that the peri-implant mucosal seal serves as a critical defense mechanism against bacterial invasion and inflammatory breakdown around implants [20]. The favourable soft tissue parameters observed around implants in the present study therefore support the concept that stable peri-implant mucosal conditions contribute to long-term implant success. Despite these encouraging findings, certain limitations must be considered. The study involved a relatively small sample size and the follow-up period was limited to 12 months. Peri-implant diseases such as peri-implantitis may develop over longer periods of functional loading. Consequently, larger randomized clinical trials with extended follow-up periods are required to confirm the long-term biological stability of single-piece implant systems. Overall, the results of this study demonstrate that single-piece implants exhibit favourable peri-implant tissue responses characterized by stable probing depths, reduced bleeding on probing, and minimal marginal bone remodeling. These findings suggest that single-piece implant systems may represent a biologically stable and predictable treatment option for single- tooth replacement when appropriate surgical protocols and prosthetic designs are employed.

## Conclusion:

Single-piece implants demonstrated comparable or superior peri-implant soft tissue health compared with natural abutment teeth supporting conventional bridges. Minimal bone remodeling and stable mucosal parameters indicate favourable biological compatibility. Future long-term studies are recommended to validate these findings over extended observation periods.
